# Aspartame may promote erectile dysfunction via DPP4-mediated endothelial dysfunction and apoptosis: evidence from network toxicology, molecular dynamics simulation, and experimental validation

**DOI:** 10.3389/fnut.2026.1841453

**Published:** 2026-06-02

**Authors:** Zhiqiang Dai, Shouqiang Wang, Zhigui Chen, Qiang Zhang, Boyi Wang

**Affiliations:** Department of Urology, Meishan City Second People's Hospital, Meishan, Sichuan, China

**Keywords:** aspartame, erectile dysfunction, food safety, network toxicology, reproductive toxicology

## Abstract

**Background:**

Aspartame is one of the most widely used artificial sweeteners, but its potential adverse effects on male reproductive health remain insufficiently understood. This study aimed to investigate the potential mechanisms by which aspartame may contribute to Erectile dysfunction (ED).

**Methods:**

Potential targets of aspartame were identified using SEA, SwissTargetPrediction, and TargetNet, while ED-related genes were collected from GeneCards. Overlapping genes were analyzed using protein–protein interaction, Gene Ontology, and Kyoto Encyclopedia of Genes and Genomes enrichment analyses. The GSE2457 dataset was used to identify differentially expressed genes, followed by LASSO regression and nomogram construction to screen key targets. Molecular dynamics simulation was performed to evaluate the stability of ligand–protein binding. Penile corpus cavernosum endothelial cells were then used for wound healing and western blot assays.

**Results:**

A total of 133 overlapping genes between aspartame and ED were identified, mainly enriched in vascular regulation, endothelial signaling, apoptosis, and PI3K-Akt/HIF-1-related pathways. Integrated analysis of GSE2457 identified 7 overlapping genes, among which DPP4, GLUL, and HRAS were selected as key genes, with DPP4 showing the strongest predictive value. Molecular dynamics simulation indicated stable binding between aspartame and DPP4. *In vitro*, aspartame impaired endothelial cell migration, increased DPP4 and Bax expression, and decreased p-eNOS/eNOS and Bcl-2 expression, whereas saxagliptin partially reversed these effects.

**Conclusion:**

Aspartame may promote ED progression by inducing endothelial dysfunction and apoptosis, with DPP4 emerging as a potential key target.

## Introduction

Aspartame is a widely used high-intensity artificial sweetener in the food industry (1). Owing to its high sweetness and low caloric content, it is extensively incorporated into sugar-free beverages, baked goods, and other low-calorie products, and has become an important sucrose substitute for controlling sugar consumption and reducing energy intake (2). Current regulatory assessments generally consider aspartame to be safe at approved or commonly encountered exposure levels. The acceptable daily intake (ADI) for aspartame has been established at 40 mg/kg body weight/day by EFSA and JECFA, while the U. S. FDA has set a higher ADI of 50 mg/kg body weight/day (3). Notably, in 2023, IARC classified aspartame as Group 2B, or “possibly carcinogenic to humans” (4). JECFA reaffirmed the ADI of 0–40 mg/kg body weight/day in the same risk assessment context. These regulatory evaluations indicate that the safety of aspartame should be interpreted in relation to exposure levels, while also highlighting the need for continued investigation into its potential biological effects under specific experimental or susceptible disease context. However, accumulating experimental evidence suggests that, under certain exposure conditions, aspartame and its metabolites may still induce oxidative stress, mitochondrial damage, and apoptosis related alterations, indicating that its potential biological effects may extend beyond its role as a mere caloric substitute (5–7).

The development of erectile dysfunction (ED) is closely associated with endothelial dysfunction and apoptosis in the penile corpus cavernosum (8). Endothelial nitric oxide synthase (eNOS) mediated nitric oxide (NO) production is a key determinant of corpus cavernosum smooth muscle relaxation and normal erectile function. Downregulation of eNOS expression or impairment of its activity can reduce NO bioavailability, weaken downstream cGMP signaling, and ultimately result in erectile impairment (9). In addition, under sustained pathological conditions such as oxidative stress, metabolic disturbances, and inflammation, excessive apoptosis of cavernosal endothelial cells and smooth muscle cells may further disrupt local tissue homeostasis and vasodilatory function (10, 11). Therefore, dysregulation of eNOS/NO signaling and aberrant apoptosis may constitute important pathological bases for the onset and progression of ED (12).

ED is a highly heterogeneous disorder that may result from vasculogenic, neurogenic, endocrine/metabolic, psychogenic, or mixed etiologies, and the predominant pathological basis may differ across ED subtypes (13). Existing evidence suggests that aspartame may be involved in oxidative stress, inflammatory responses, mitochondrial dysfunction, apoptosis, and endothelial injury, all of which are particularly relevant to vasculogenic and metabolic ED (5, 7). Diabetic erectile dysfunction (DMED) represents a typical metabolic/vasculogenic ED phenotype, characterized mainly by corpus cavernosum endothelial dysfunction, impaired eNOS/NO/cGMP signaling, enhanced oxidative stress, and increased apoptosis (14). Therefore, in the present study, a DMED-related dataset was selected as a representative disease model to investigate whether aspartame-related targets may be enriched in ED mechanisms associated with endothelial injury and apoptosis.

At present, direct evidence linking aspartame to ED remains limited. Existing studies suggest that aspartame can induce oxidative stress, inflammatory responses, and apoptosis, and may impair endothelial function and reduce NO bioavailability, thereby potentially affecting key pathological processes involved in ED (15). However, its specific molecular targets and mechanisms of action remain unclear.

**Figure 1:**
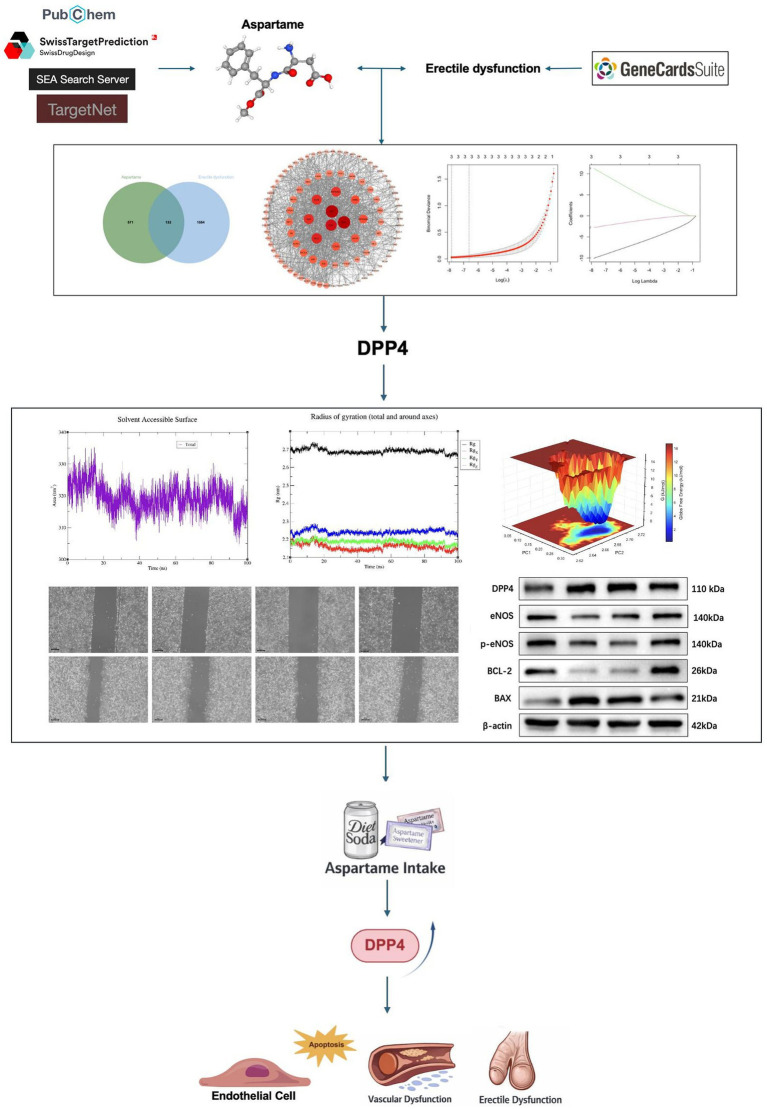
Overall workflow of the study.

Given that the toxic effects of exogenous chemicals are often characterized by multi-target and multi-pathway interactions, network toxicology can be used to systematically identify potential overlapping targets and key signaling pathways between aspartame and ED. Molecular docking can further predict the binding affinity of aspartame to core target proteins, while molecular dynamics simulation can evaluate the dynamic stability of ligand–receptor interactions (16, 17). Accordingly, this study aims to integrate network toxicology, molecular docking, and molecular dynamics simulation to systematically explore the potential targets, key signaling pathways, and molecular interaction mechanisms through which aspartame may influence ED, thereby providing a theoretical basis for further elucidating its possible toxic effects. A detailed research workflow is shown in Figure 1.

## Methods

### Toxicity prediction

First, the keyword “aspartame” was searched in the PubChem database to obtain the SMILES string and the three-dimensional SDF structure file of aspartame. Subsequently, following previously reported methods (18), three online platforms, ProTox-3.0, ADMETlab 3.0, and AdmetSAR 3.0, were used to predict the toxicity profile of aspartame. ProTox-3.0 and ADMETlab 3.0 were mainly applied to predict common human health related toxicities, whereas AdmetSAR 3.0 was further used to evaluate reproductive toxicity.

### Identification of aspartame- and ED related targets

According to previously reported methods (19), the chemical structure of aspartame obtained in the previous step was uploaded to the SEA, TargetNet, and SwissTargetPrediction databases to predict its potential target genes (20, 21).

For SEA and SwissTargetPrediction, the species was restricted to *Homo sapiens*, and all predicted target genes were downloaded. If the same gene symbol appeared repeatedly, only the first occurrence was retained. For TargetNet, the default screening criteria were applied: AUC > 0.7, fingerprint type = ECFP4 fingerprints, and predicted probability > 0.6. Because TargetNet outputs target genes in UniProt ID format, the resulting UniProt IDs were further converted into standard gene symbols using the UniProt database. The prediction results from these three databases were then merged and de-duplicated to obtain the final set of potential target genes of aspartame (22).

The keyword “erectile dysfunction” was searched in the GeneCards database, and the top 50% of genes ranked by relevance score were selected as ED related genes. This screening strategy was adopted based on previous network toxicology studies, which commonly use the upper 50% of GeneCards-ranked genes as disease-related targets to enhance target relevance and reduce potential false-positive interference from genes with relatively low relevance scores (23, 24).

The intersection between the potential target genes of aspartame and the ED related genes was then identified to obtain the preliminary aspartame–ED related genes.

### PPI network construction

The screened aspartame–ED related genes were imported into the STRING online database to construct a protein–protein interaction (PPI) network (25). The species was limited to *Homo sapiens*, the interaction confidence score was set at ≥0.4, and disconnected nodes were hidden. This cutoff corresponds to the “medium confidence” level recommended by STRING and is commonly adopted in network toxicology studies for preliminary PPI screening, as it balances network size with interaction reliability (23).

The resulting PPI network was then visualized and analyzed using Cytoscape 3.10. Topological parameters of each node were calculated with the Network Analyzer plugin. Among these parameters, the degree value represents the number of connections between a given node and other nodes and can therefore be used to evaluate its centrality and potential biological importance within the network. In general, a higher degree value indicates that the gene is involved in more extensive interactions and may play a more critical regulatory role in the relevant biological processes. Finally, all nodes were ranked according to degree values to identify potential hub genes.

### Enrichment analysis

To further elucidate the potential biological functions of the aspartame–ED related genes and the molecular regulatory networks in which they may participate, Gene Ontology (GO) functional annotation and Kyoto Encyclopedia of Genes and Genomes (KEGG) pathway enrichment analyses were performed using the clusterProfiler package in R. For GO enrichment analysis, the enrichGO function was used with the org. Hs.eg.db package as the human gene annotation database. Gene symbols were used as the input identifier, and the ontology parameter was set to “ALL” to include biological process (BP), molecular function (MF), and cellular component (CC) categories.

For KEGG pathway enrichment analysis, gene symbols were first converted into Entrez IDs using the bitr function in clusterProfiler with org. Hs.eg.db as the annotation database. KEGG enrichment analysis was then conducted using the enrichKEGG function, with the organism set to *Homo sapiens* (“hsa”). The enriched KEGG results were subsequently converted back into readable gene symbols using the setReadable function.

Enrichment significance was evaluated using a hypergeometric test, and multiple testing correction was performed using the Benjamini–Hochberg method. Adjusted *p* value or q value was used as the criterion for significance. The top 10 significantly enriched GO terms and KEGG pathways were finally selected for visualization using bar plots and dot plots.

### Processing of the ED dataset

The GSE2457 dataset was downloaded from the NCBI Gene Expression Omnibus (GEO) database for subsequent bioinformatics analyses. This dataset contains gene expression microarray data related to diabetic erectile dysfunction, generated on the GPL341 platform, and includes 10 corpus cavernosum tissue samples, comprising 5 normal control samples and 5 diabetic ED (DMED) samples (26). The DMED model was established using corpus cavernosum tissue from F344 rats 10 weeks after streptozotocin-induced diabetes.

After downloading the raw expression matrix and the corresponding platform annotation file, probe IDs were first converted into their corresponding gene symbols. When multiple probes mapped to the same gene, the average expression value was used as the final expression level of that gene. Probes without a clearly annotated gene symbol were removed, yielding a standardized gene expression matrix for downstream analyses. Finally, to ensure consistency with previous analyses and facilitate subsequent integrative analysis, rat gene IDs were converted into their human homologs using the “msigdbr” package.

Differential expression analysis was performed using the limma package in R. For each gene, the log2 fold change (log2FC), *t* statistic, and corresponding *p* value were calculated. According to the method reported by Sullivan CJ et al., genes with *p* < 0.05 and |log2FC| > 0.585 were considered differentially expressed, where |log2FC| > 0.585 corresponds to a greater than 1.5-fold change in gene expression (26).

After identifying the differentially expressed genes (DEGs), a volcano plot was generated to visualize the differential expression profile. This plot simultaneously reflects both the magnitude of expression changes and their statistical significance. Upregulated and downregulated genes were displayed in different colors, and the most significantly altered genes were labeled to provide a more intuitive overview of the DMED related gene expression pattern.

### Machine learning analysis

The DEGs were intersected with the previously identified aspartame–ED related genes to screen candidate genes associated with both abnormal expression in DMED and the effects of aspartame. Based on these overlapping genes, a feature gene expression matrix was constructed, and least absolute shrinkage and selection operator (LASSO) regression was further applied for feature selection. LASSO analysis was performed using the glmnet package in R, and the optimal penalty parameter *λ* was determined by cross-validation, thereby identifying the key genes most closely associated with the DMED phenotype.

### Nomogram construction

Based on the feature genes selected by LASSO regression, a logistic regression model was further used to construct a nomogram prediction model. First, the expression matrix of the selected genes in each sample was extracted, and group status was defined as the dependent variable, with the experimental group coded as 1 and the control group coded as 0. Logistic regression analysis was then performed using the lrm function in the rms package in R to establish the disease risk prediction model. On this basis, the regression model was visualized using the nomogram function, and a nomogram was generated by assigning a corresponding score to each predictor and summing these scores to estimate the individual probability of disease occurrence.

To evaluate the predictive performance of the nomogram model, a calibration curve was further plotted. Internal validation was performed using bootstrap resampling to compare the predicted probabilities with the observed outcomes. A calibration curve closer to the ideal diagonal line indicates better agreement between the predicted and actual outcomes. All analyses were conducted in R, and a two-sided *p* < 0.05 was considered statistically significant.

### Molecular docking

Molecular docking was performed using AutoDock Vina under a semi-flexible docking protocol, in which the receptor was treated as rigid and the ligand was treated as fully flexible, to balance computational cost and predictive performance. Three-dimensional protein structures were obtained from the RCSB PDB database, and the three-dimensional ligand structure of aspartame was downloaded from the PubChem database. The docking grid box was defined to cover the entire protein to minimize bias toward a predefined binding pocket and to allow unbiased exploration of potential binding sites. The Vina parameters were set as follows: exhaustiveness = 8, num_modes = 10, and energy range = 3. For each protein–ligand pair, 10 independent docking runs were performed using different random seeds, and the conformation with the lowest Vina score was selected for subsequent interaction analysis. A Vina score < −5.0 kcal/mol was used as an operational criterion for relatively stable binding. Finally, the docking conformations and key interactions were visualized using PyMOL.

### Molecular dynamics simulation

All-atom molecular dynamics (MD) simulations were performed using GROMACS. The ligand was first preprocessed using AmberTools 22 and parameterized based on the general AMBER force field (GAFF). Hydrogen atoms were then added, and restrained electrostatic potential (RESP) charges were calculated using Gaussian 16 W, based on which the ligand topology file was generated. The protein was parameterized using the AMBER99SB-ILDN force field. Each system was solvated in the SPC water model, and Na^+^/Cl^−^ ions were added to neutralize the system. The system then underwent energy minimization using the steepest descent and conjugate gradient methods. After energy minimization, the system was equilibrated sequentially under the NVT and NPT ensembles, followed by a 100 ns MD simulation at 300 K and 1 bar.

After the simulation, the structural stability of the complex was evaluated by analyzing root mean square deviation (RMSD), root mean square fluctuation (RMSF), radius of gyration (Rg), solvent-accessible surface area (SASA), and the number/occupancy of hydrogen bonds. In addition, a free energy landscape (FEL) was constructed to assess the conformational convergence of the system and the rationality of its dominant conformational states.

### Cell culture and treatment

Saxagliptin is a highly selective and potent DPP4 inhibitor. Previous studies have shown that 0.5 μmol/L saxagliptin is sufficient to achieve the maximal inhibitory effect on DPP4 *in vitro* in HUVECs; therefore, 0.5 μmol/L was selected as the intervention concentration in the present study (27, 28). Aspartame (purity ≥ 99%; Solarbio) was dissolved in dimethyl sulfoxide to prepare a stock solution and then further diluted in complete culture medium to the desired working concentrations. Cells were treated with different concentrations of aspartame for 48 h. To minimize the potential influence of compound degradation, cellular metabolism, or interference from culture medium components during the exposure period, the culture medium was replaced after 24 h with freshly prepared complete medium containing the same concentration of aspartame.

Based on the RT-qPCR results from the concentration gradient experiment, 2.0 μM aspartame was selected for subsequent cell intervention experiments, as CCECs remained in a normal growth state at this concentration while showing the most pronounced induction of DPP4 expression.

Penile corpus cavernosum endothelial cells (CCECs; catalog no. CP-R133) were purchased from Procell (China). Cells were cultured in EBM-2 medium (Lonza, Switzerland) supplemented with fetal bovine serum and penicillin/streptomycin under standard conditions at 37 °C in a humidified incubator with 5% CO₂. Cells in good growth condition and at approximately 80% confluence were used for subsequent experiments. Before treatment, cells were seeded into 6-well plates at a density of 2 × 10^5^ cells/well. According to the experimental design, the cells were randomly divided into four groups: the normal control group (NC), cultured in regular medium; the high-glucose group (HG), treated with high-glucose medium with a final glucose concentration of 35 mmol/L; the Aspartame group, treated with aspartame; and the Aspartame + Saxagliptin group, treated with aspartame in combination with saxagliptin.

### RT-qPCR

Total RNA was extracted from CCECs using the Foregene Total RNA Extraction Kit (Cat# RE-03111, China), and RNA concentration was measured using a NanoDrop spectrophotometer (Thermo Scientific, United States). Subsequently, 1 μg of total RNA was reverse transcribed into cDNA using the ReverTra Ace qPCR RT Master Mix (Toyobo, Japan).

Quantitative real-time PCR was performed using SYBR Green qPCR Master Mix in a total reaction volume of 10 μL. The primer sequences for DPP4 were as follows: forward, 5′-ATTCCGTACCCAAAGGCAGG-3′; reverse, 5′-AGGCCACGTCACACAAGTAG-3′. GAPDH was used as the internal control, with the following primer sequences: forward, 5′-GCATCTTCTTGTGCAGTGCC-3′; reverse, 5′-TACGGCCAAATCCGTTCACA-3′. Amplification was carried out on an ABI 7500 real-time PCR system (Thermo Scientific, United States) under the following cycling conditions: 95 °C for 60 s, followed by 35 cycles of 95 °C for 15 s, 65 °C for 30 s, and 72 °C for 30 s. The relative expression level of DPP4 was calculated using the 2^−ΔΔCt^ method and normalized to GAPDH.

### Wound healing assay

A wound healing assay was performed to evaluate the migratory ability of CCECs under different treatment conditions. Cells were seeded in 6-well plates and allowed to grow to 90–100% confluence. A straight scratch was then created vertically across the center of the cell monolayer using a sterile 200 μL pipette tip. The wells were gently washed twice with phosphate-buffered saline to remove detached cells, followed by replacement with serum-free medium under the corresponding treatment conditions. Cells were then subjected to normal culture, high glucose, aspartame, or aspartame plus saxagliptin treatment according to group assignment. Images of the wound area were captured under an inverted microscope at 0 h and 48 h. The acquired images were quantitatively analyzed using ImageJ software. Specifically, the wound width or cell-free area at 0 h and 48 h was measured for each group, and the relative wound closure rate was calculated as follows: wound closure rate = (initial wound area − remaining wound area) / initial wound area × 100%, which was used to reflect the migratory repair ability of the cells.

### Total protein extraction and western blotting

Total proteins were extracted from CCECs in each group using a total protein extraction kit (Solarbio, China). During lysis, 1% protease inhibitor and 1% phosphatase inhibitor were added to the lysis buffer to minimize protein degradation and dephosphorylation. The entire extraction procedure was performed on ice to maintain low-temperature conditions and reduce the risk of protein denaturation. Protein concentrations were subsequently measured using a BCA protein assay kit (Thermo Scientific, United States) according to the manufacturer’s instructions to ensure equal sample loading.

Equal amounts of protein were separated by SDS-PAGE and transferred onto membranes. Electrophoresis was performed at 60 V for 30 min, followed by 120 V for 60 min, to obtain satisfactory protein separation. Proteins were then transferred onto 0.45 μm PVDF membranes at a constant current of 400 mA for 30 min. After transfer, the membranes were blocked with rapid blocking solution (Epizyme Biotech, China) for 15 min at room temperature and washed with TBST buffer. The PVDF membranes were then incubated overnight at 4 °C with the following primary antibodies: DPP4 (Proteintech, China, 1:1000), Bcl-2 (Proteintech, China, 1:5000), Bax (Proteintech, China, 1:5000), p-eNOS (Cell Signaling Technology, United States, 1:1000), eNOS (Cell Signaling Technology, United States, 1:1000) and β-actin (Proteintech, China, 1:10000).

On the following day, the membranes were thoroughly washed with TBST buffer to remove unbound primary antibodies. They were then incubated for 1 h at room temperature with horseradish peroxidase (HRP)-conjugated goat anti-rabbit or goat anti-mouse secondary antibodies (Easybio, China). After secondary antibody incubation, the membranes were washed again with TBST buffer to reduce nonspecific binding. Protein bands were visualized using a chemiluminescence imaging system (Tanon, China), and band intensities were quantified using ImageJ software to determine the relative expression levels of the target proteins.

### Flow cytometric analysis of apoptosis and caspase-3 expression

Apoptosis of CCECs was evaluated using an Annexin V-FITC/PI apoptosis detection kit (Lianke Bio, China; Cat. No. 70-AP101) according to the manufacturer’s instructions. After the indicated treatments, cells from each group were collected and washed twice with PBS. The 5 × Binding Buffer supplied with the kit was diluted with double-distilled water to prepare 1 × Binding Buffer, and the cells were resuspended in 500 μL of 1 × Binding Buffer. Blank and single-stained control tubes were prepared simultaneously for instrument setup and compensation. The blank control received no staining reagent; the two single-stained controls were incubated with 5 μL Annexin V-FITC or 10 μL propidium iodide (PI), respectively; and the sample tubes were stained with both 5 μL Annexin V-FITC and 10 μL PI. After gentle vortex mixing, the cells were incubated for 5 min at room temperature in the dark. Flow cytometric analysis was then performed using an Attune NxT flow cytometer (Thermo Fisher Scientific, United States). Instrument voltage was adjusted using the blank control, and fluorescence compensation was set using the single-stained controls. Annexin V-FITC was detected in the BL-1 channel, and PI was detected in the BL-2 channel. The percentages of apoptotic cells were analyzed based on the distribution of Annexin V-FITC/PI staining.

Caspase-3 expression in CCECs was assessed using the FLICA660 caspase-3 fluorescent probe according to the manufacturer’s instructions. After the indicated treatments, cells from each group were collected and adjusted to a density of approximately 2 × 10^5^ cells/mL. The FLICA660 probe was dissolved in 50 μL DMSO and further diluted with PBS to prepare a 30 × working solution. Meanwhile, 1 × cell apoptosis staining buffer was prepared for subsequent washing steps. For staining, 10 μL of the FLICA660 working solution was added to 290 μL of the cell suspension to obtain a final volume of 300 μL, followed by gentle vortex mixing. The cells were then incubated at 37 °C for 1 h in the dark, with gentle vortexing every 10–20 min to ensure sufficient contact between the cells and the fluorescent probe. After incubation, the cells were centrifuged, washed twice with cell apoptosis staining buffer, resuspended, and then analyzed by flow cytometry. The fluorescence intensity of caspase-3 staining was used to evaluate the change in caspase-3 expression in each group.

### Statistical analysis

Statistical analyses were performed using GraphPad Prism 10 (GraphPad Software, United States). Gray values of western blot bands were semi-quantified using ImageJ software and normalized to β-actin. All data are presented as mean±standard deviation. Normality was assessed using the Shapiro–Wilk test, and homogeneity of variance was evaluated using the Brown–Forsythe test. For data that were normally distributed and met the assumption of homogeneity of variance, one-way analysis of variance (ANOVA) was used, followed by prespecified linear contrast tests for between-group comparisons. For data with unequal variances, Welch’s ANOVA followed by the Games–Howell *post hoc* test was applied. For data that did not meet the assumptions for parametric testing, the Kruskal–Wallis test followed by Dunn’s *post hoc* test was used for multiple-group comparisons. All *p* values were two-sided, and *p* < 0.05 was considered statistically significant.

## Results

### Toxicity profile of aspartame

**Figure 2:**
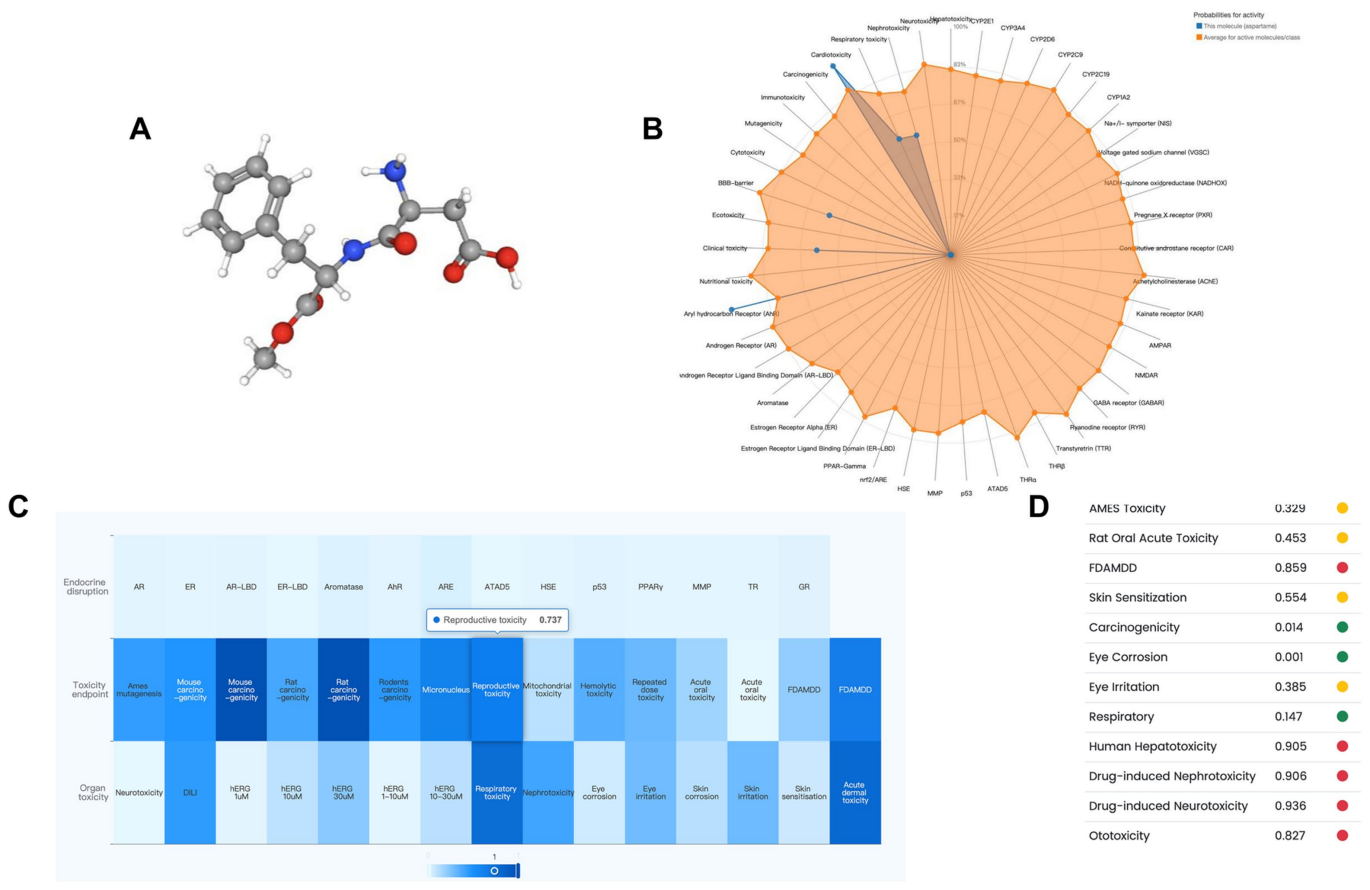
Toxicity prediction profile of aspartame. **(A)** Chemical structure of aspartame obtained from the PubChem database. **(B)** Toxicity prediction of aspartame generated using the ProTox-3.0 platform. **(C)** Toxicity heatmap of aspartame predicted by AdmetSAR 3.0 platform. **(D)** Toxicity prediction of aspartame generated using the ADMETlab 3.0 platform.

The SMILES information and chemical structure of aspartame were obtained from the PubChem database (Figure 2A). Toxicity prediction using the ProTox-3.0 database suggested that aspartame may be associated with nephrotoxicity, respiratory toxicity, cardiotoxicity, and clinical toxicity. In addition, it may also be associated with activation of the aryl hydrocarbon receptor (AhR) (Figure 2B).

Predictions from the AdmetSAR 3.0 database showed that aspartame may exhibit respiratory toxicity, nephrotoxicity, acute dermal toxicity, carcinogenicity, reproductive toxicity, and hemolytic toxicity (Figure 2C). Notably, the predicted results for repeated-dose toxicity and biodegradability suggested that its toxic effects may be related to dose dependence and poor biodegradability.

Further analysis using ADMETlab 3.0 showed that aspartame may also be associated with multiple toxic effects, including acute oral toxicity in rats, skin sensitization, eye irritation, human hepatotoxicity, drug-induced nephrotoxicity, drug-induced neurotoxicity, and ototoxicity (Figure 2D).

### Aspartame–ED related genes

**Figure 3:**
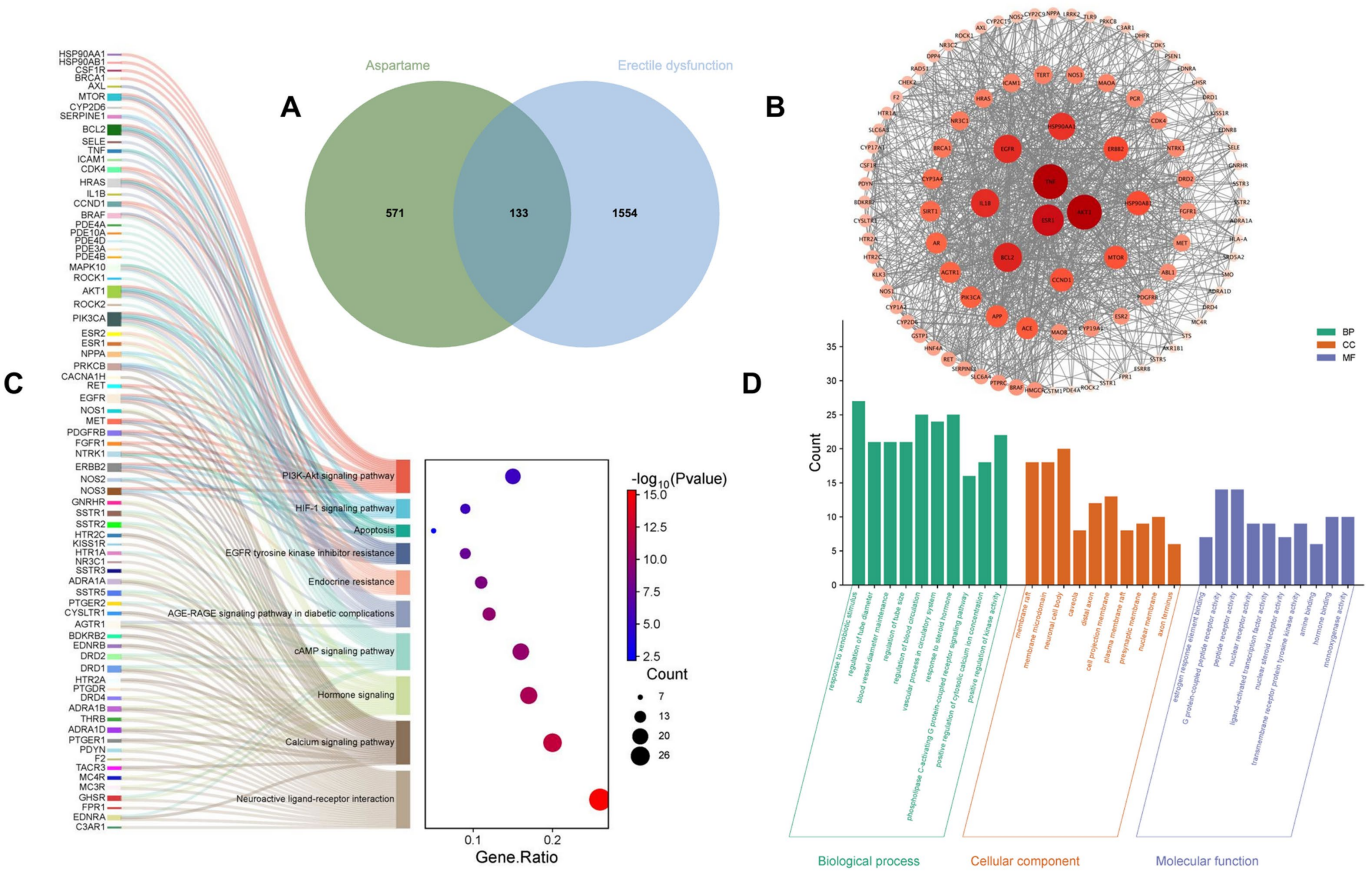
Identification of shared targets between aspartame and erectile dysfunction and functional enrichment analysis. **(A)** Venn diagram showing the overlapping target genes between aspartame and erectile dysfunction (ED). **(B)** Protein–protein interaction (PPI) network of the overlapping genes. Node size and color intensity represent the degree of connectivity. **(C)** KEGG enrichment analysis of the overlapping genes. The left panel shows the gene–pathway association network, and the right panel shows the bubble plot of the top enriched pathways. **(D)** GO enrichment analysis of the overlapping genes, including biological process (BP), cellular component (CC), and molecular function (MF) categories.

A total of 704 potential target genes of aspartame and 1,687 ED related genes were identified. Intersection analysis yielded 133 overlapping genes (Figure 3A). Further construction of the protein interaction network revealed that genes such as TNF, AKT1, ESR1, and BCL2 occupied central positions in the network, with relatively high connectivity and potential regulatory importance (Figure 3B). These findings suggest that such key nodes may serve as bridges linking aspartame related toxicity to the pathological processes of ED. Collectively, these results indicate that aspartame may participate in the development of ED by regulating multiple biological processes, including inflammatory responses, cell survival and apoptosis, hormone signaling, and vascular endothelial function.

### KEGG enrichment analysis

KEGG pathway enrichment analysis showed that these genes were mainly enriched in the PI3K-Akt signaling pathway, HIF-1 signaling pathway, apoptosis, AGE-RAGE signaling pathway in diabetic complications, cAMP signaling pathway, calcium signaling pathway, and neuroactive ligand-receptor interaction. Among these, the significant enrichment of the apoptosis pathway suggests that apoptosis may represent one of the key mechanisms by which aspartame contributes to ED (Figure 3C). Combined with the finding that BCL2, TNF, and AKT1 occupied central positions in the PPI network, this result indicates that aspartame may enhance programmed cell death by disrupting the balance between pro-apoptotic and anti-apoptotic molecules, thereby impairing the stability of the corpus cavernosum microenvironment. In addition, the significant enrichment of the PI3K-Akt and HIF-1 signaling pathways further supports the possibility that aspartame may mediate tissue injury by affecting cell survival, oxidative stress, and hypoxia responses. The enrichment of the AGE-RAGE signaling pathway in diabetic complications also suggests that aspartame related targets are closely linked to the molecular networks involved in diabetic complications. Meanwhile, enrichment of the cAMP and calcium signaling pathways indicates that aspartame may also aggravate ED by disturbing signaling pathways related to vasomotion and smooth muscle relaxation.

### GO enrichment analysis

At the biological process level, the overlapping genes were mainly enriched in response to xenobiotic stimulus, regulation of tube diameter, maintenance of blood vessel diameter, regulation of blood circulation, regulation of vasculature development, response to steroid hormone, G protein-coupled receptor signaling, positive regulation of cytosolic calcium ion concentration, and positive regulation of kinase activity. These results suggest that aspartame related target genes may be primarily involved in the regulation of vascular tone, maintenance of circulatory homeostasis, and abnormalities in intracellular signal transduction. Because the maintenance of erectile function relies heavily on the integrity of the cavernosal endothelium and adequate blood perfusion, these enriched terms imply that aspartame may contribute to the development and progression of ED by interfering with vasomotor function and endothelial signaling, thereby inducing endothelial dysfunction.

At the cellular component level, the overlapping genes were mainly enriched in membrane rafts, membrane microdomains, caveolae, plasma membrane, cell projection membrane, and nuclear membrane, as well as in neuronal cell bodies, distal axons, presynaptic membranes, and axon terminals. Notably, membrane rafts and caveolae serve as important platforms for the clustering and transduction of various receptors, kinases, and endothelial signaling molecules, suggesting that these shared target genes may mediate vascular endothelial injury and related dysfunction by affecting receptor complexes and the endothelial signaling microenvironment.

At the molecular function level, the overlapping genes were primarily enriched in G protein-coupled peptide receptor activity, peptide receptor activity, nuclear receptor activity, ligand-activated transcription factor activity, steroid hormone receptor activity, transmembrane receptor protein tyrosine kinase activity, hormone binding, and amine binding. These findings indicate that the shared target genes are broadly involved in receptor-mediated recognition of extracellular stimuli and amplification of intracellular signaling cascades, suggesting that aspartame may further affect endothelial homeostasis and cell fate regulation by disrupting receptor-dependent signaling networks.

Overall, GO enrichment analysis indicated that the shared targets of aspartame and ED were mainly associated with vascular homeostasis imbalance, abnormal endothelial signal transduction, and enhanced cellular stress responses, thereby providing functional support for the hypothesis that aspartame may induce endothelial injury (Figure 3D).

### Integrated analysis of the ED dataset

**Figure 4:**
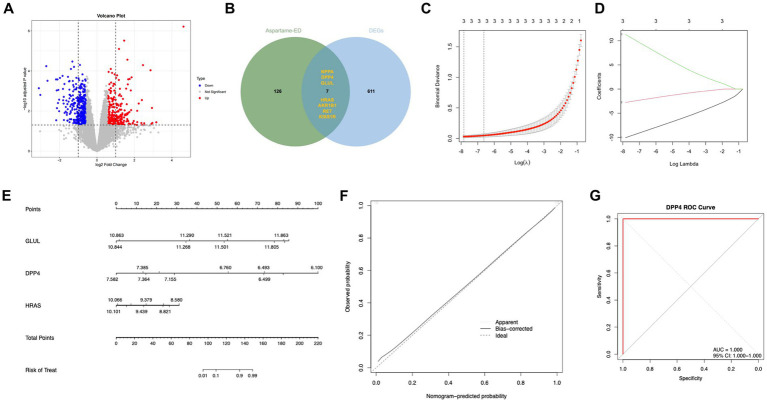
Integrated bioinformatics and machine learning analyses for key target screening. **(A)** Volcano plot of differentially expressed genes (DEGs) in the GSE2457 dataset. Red dots indicate upregulated genes, blue dots indicate downregulated genes, and gray dots indicate genes without significant differential expression. **(B)** Venn diagram showing the intersection between aspartame–ED related genes and DEGs from the GSE2457 dataset. **(C)** Cross-validation curve of the LASSO regression model used to identify optimal feature genes. **(D)** LASSO coefficient profiles of candidate genes. **(E)** Nomogram model constructed based on the selected key genes (GLUL, DPP4, and HRAS). **(F)** Calibration curve of the nomogram model, showing the agreement between predicted and observed probabilities. **(G)** Receiver operating characteristic (ROC) curve of DPP4 in the GSE2457 dataset, showing its discriminatory performance between the control and disease groups.

In the GSE2457 dataset, a total of 618 DEGs were identified. After intersecting these DEGs with the previously obtained aspartame–ED related target genes, 7 overlapping genes were identified, namely NPPA, DPP4, GLUL, HRAS, AKR1B1, RET, and KISS1R (Figures 4A,B). These results suggest that these genes may represent important candidate targets linking aspartame exposure to the development and progression of ED.

To further identify representative key molecules among the overlapping genes, LASSO regression was used for feature compression and variable selection (Figures 4C,D). The results showed that, with changes in the penalty parameter *λ*, the coefficients of some variables gradually shrank to zero, and ultimately three key genes with the greatest diagnostic value were retained: DPP4, GLUL, and HRAS. This finding suggests that these three genes are highly representative in distinguishing DMED samples from control samples and may play important roles in the pathological process through which aspartame contributes to ED.

Based on these key genes, a nomogram prediction model was further constructed (Figure 4E). The results showed that all three genes contributed to the model risk score, with DPP4 making a relatively greater contribution, suggesting that it has strong discriminatory power for predicting ED risk and may be a core target deserving particular attention in the process by which aspartame promotes ED progression. The calibration curve further demonstrated good agreement between the predicted and observed outcomes, indicating that the constructed model had good fit and predictive performance (Figure 4F). In addition, the ROC curve for DPP4 yielded an AUC of 1 in this small-sample sequencing dataset (Figure 4G), suggesting excellent discriminatory performance between the control and disease groups, although this result should be interpreted with caution given the limited sample size.

### Molecular docking

**Figure 5:**
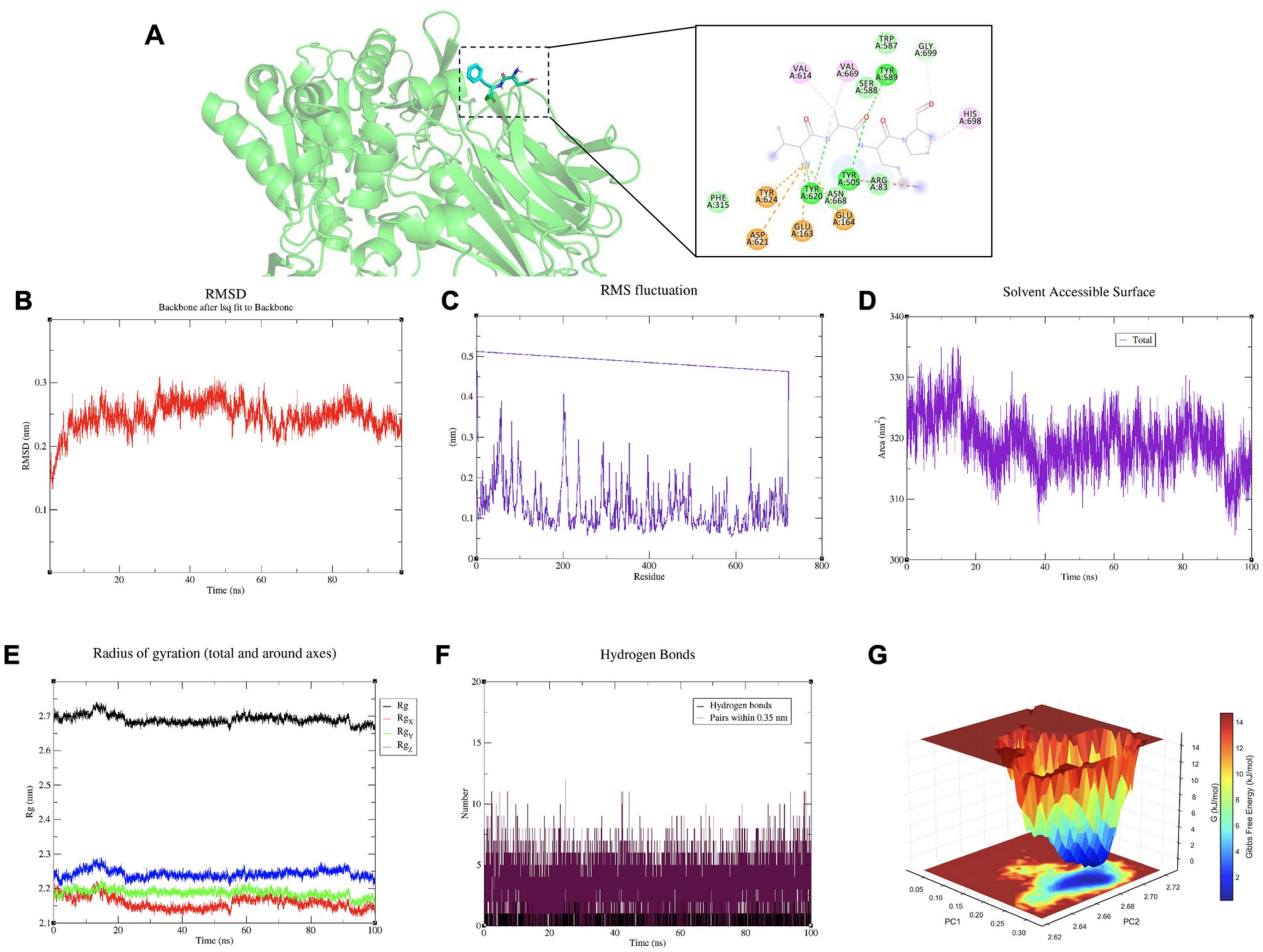
Molecular dynamics simulation of the aspartame–DPP4 complex. **(A)** Molecular docking conformation and two-dimensional interaction diagram of aspartame bound to DPP4. **(B)** Root mean square deviation (RMSD) of the complex during the 100 ns simulation. **(C)** Root mean square fluctuation (RMSF) of amino acid residues. **(D)** Solvent-accessible surface area (SASA) of the complex. **(E)** Radius of gyration (Rg) of the complex. **(F)** Hydrogen bond analysis of the aspartame–DPP4 complex. **(G)** Free energy landscape (FEL) of the complex.

Molecular docking analysis demonstrated that aspartame bound to DPP4 with a binding energy of −6.7 kcal/mol, indicating relatively stable binding (Figure 5A). The docking model showed that aspartame fitted well into the active cavity of DPP4 and formed multiple interactions with surrounding amino acid residues, including Tyr620, Tyr589, Ser588, Trp587, Asn668, Arg83, Val614, and Val669. The two-dimensional interaction diagram further suggested that hydrogen bonding and hydrophobic contacts jointly contributed to stabilization of the aspartame–DPP4 complex.

### Molecular dynamics simulation

To further evaluate the stability of the interaction between aspartame and DPP4, molecular dynamics simulation of the complex was performed. The RMSD increased rapidly during the initial stage of the simulation and then gradually reached a relatively stable plateau, indicating that the aspartame–DPP4 complex underwent early conformational adjustment and subsequently maintained good overall structural stability (Figure 5B). RMSF analysis showed that most residues exhibited relatively low fluctuation amplitudes, with only a few local regions displaying higher peaks, suggesting that ligand binding did not induce marked abnormal flexibility in the overall protein structure (Figure 5C).

The SASA of the complex fluctuated within a relatively narrow range throughout the simulation and showed only a mild decrease near the end, indicating that solvent exposure remained generally stable without obvious conformational expansion or unfolding (Figure 5D). In addition, the radius of gyration remained essentially stable, with only minor fluctuations in each axis parameter, suggesting that the overall compactness of the protein was well maintained during the simulation (Figure 5E). Hydrogen bond analysis showed that a certain number of hydrogen bonds were continuously maintained between aspartame and DPP4, although dynamic fluctuations were observed, indicating that hydrogen bonding contributed to stabilization of the ligand–protein complex (Figure 5F). Furthermore, the free energy landscape exhibited relatively concentrated low-energy conformational regions, suggesting that the aspartame–DPP4 complex could stably occupy dominant conformational states and possessed favorable thermodynamic stability (Figure 5G).

### RT-qPCR

**Figure 6:**
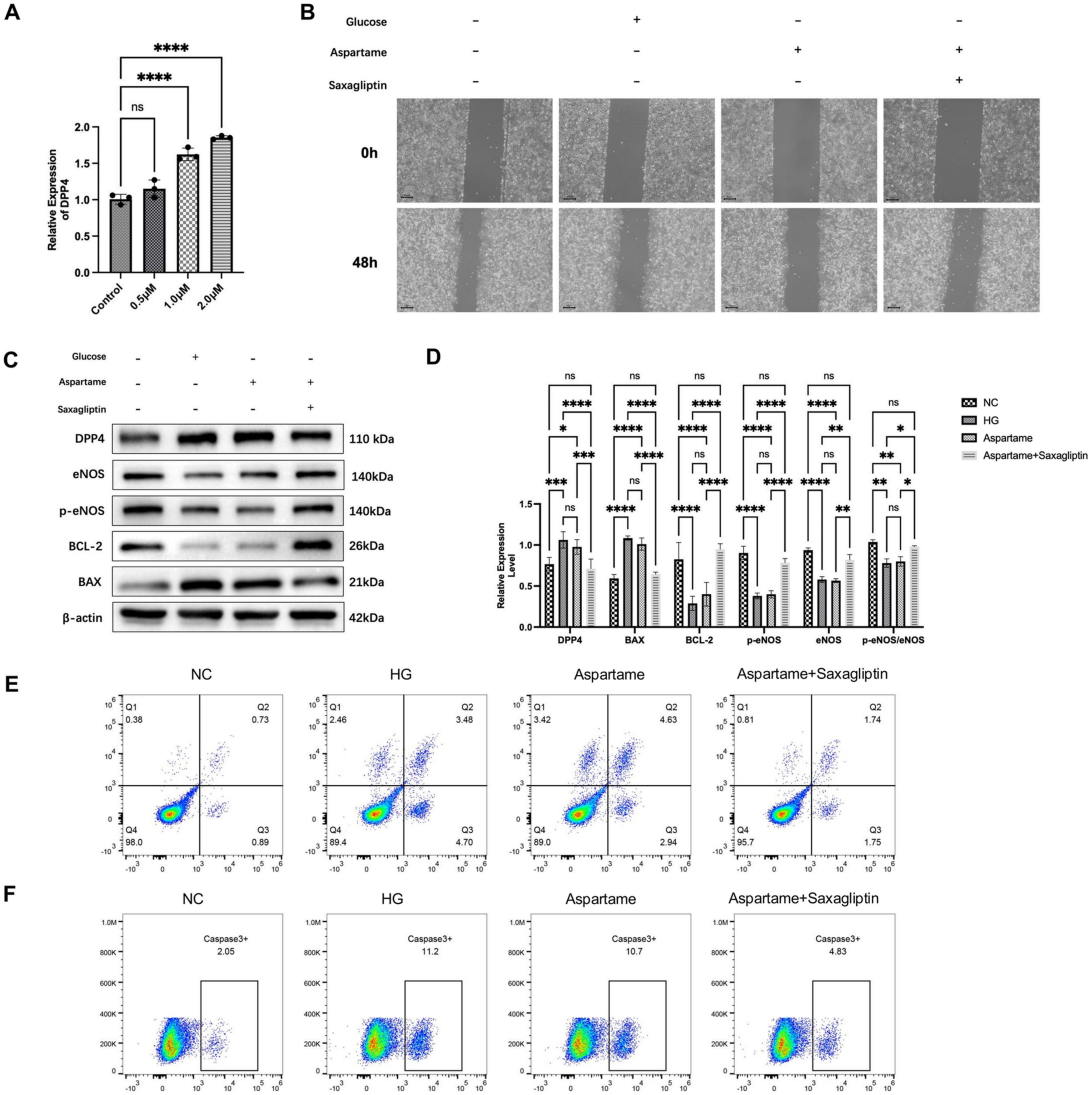
Effects of aspartame on migration, endothelial function, apoptosis, and caspase-3 expression in corpus cavernosum endothelial cells. **(A)** RT-qPCR analysis of DPP4 mRNA expression in CCECs treated with different concentrations of aspartame. ns, Not significant; *p < 0.05, **p < 0.01, ***p < 0.001, ****p < 0.0001. **(B)** Representative images of the wound healing assay in the NC, HG, Aspartame, and Aspartame + Saxagliptin groups at 0 h and 48 h. **(C)** Representative western blot bands of DPP4, p-eNOS, eNOS, Bcl-2, Bax, and β-actin in each group. **(D)** Quantitative analysis of DPP4, Bax, Bcl-2, p-eNOS, eNOS and p-eNOS/eNOS protein expression. Data are presented as mean ± SD. ns, Not significant; *p < 0.05, **p < 0.01, ***p < 0.001, ****p < 0.0001. **(E)** Flow cytometric analysis of apoptosis in CCECs from the NC, HG, Aspartame, and Aspartame + Saxagliptin groups using Annexin V-FITC/PI staining. **(F)** Flow cytometric analysis of caspase-3 expression in CCECs from the NC, HG, Aspartame, and Aspartame + Saxagliptin groups.

RT-qPCR analysis showed that DPP4 mRNA expression in CCECs exhibited an overall concentration-dependent increase after aspartame exposure (Figure 6A). Compared with the control group, treatment with 0.5 μM aspartame caused only a slight increase in DPP4 expression without statistical significance, whereas 1.0 μM and 2.0 μM aspartame significantly upregulated DPP4 mRNA levels. Among these groups, the highest DPP4 expression was observed in the 2.0 μM aspartame group.

### Wound healing assay

To evaluate the effect of aspartame on the migratory capacity of corpus cavernosum endothelial cells, wound healing assays were performed to assess wound closure at 0 h and 48 h in each group (Figure 6B). The results showed that the scratch gap in the NC group was markedly reduced after 48 h, indicating good migratory repair capacity. In contrast, both the HG group and the Aspartame group showed a lower degree of wound closure than the NC group at 48 h, suggesting that both high-glucose stimulation and aspartame treatment inhibited the migration of corpus cavernosum endothelial cells to varying degrees. Further observation showed that, compared with the Aspartame group, the Aspartame + Saxagliptin group exhibited a markedly narrower wound width and greater wound closure at 48 h, suggesting that saxagliptin intervention could partially restore the impaired endothelial cell migration induced by aspartame.

### Western blot analysis

To further verify whether aspartame mediates corpus cavernosum endothelial injury and apoptosis through a DPP4 related pathway, the protein expression levels of DPP4, p-eNOS/eNOS, Bcl-2, and Bax were measured in each group. The results showed that, compared with the NC group, DPP4 protein expression was markedly increased in both the HG and Aspartame groups. Meanwhile, the expression of p-eNOS/eNOS, an important marker of endothelial function, was significantly decreased in both the HG and Aspartame groups, indicating that both high glucose and aspartame impaired endothelial cell function (Figures 6C,D). Compared with the Aspartame group, the Aspartame + Saxagliptin group showed a clear decrease in DPP4 expression and a significant recovery in p-eNOS/eNOS expression, suggesting that inhibition of DPP4 activity could partially ameliorate aspartame-induced endothelial dysfunction. Regarding apoptosis related proteins, compared with the NC group, the pro-apoptotic protein Bax was significantly upregulated, whereas the anti-apoptotic protein Bcl-2 was markedly downregulated in both the HG and Aspartame groups, indicating that the cells were in a pro-apoptotic state. After saxagliptin intervention, Bax expression decreased and Bcl-2 expression increased in the Aspartame + Saxagliptin group, suggesting that DPP4 inhibition could effectively alleviate the apoptosis imbalance induced by aspartame.

### Flow cytometric analysis of apoptosis and caspase-3 expression

Flow cytometric analysis showed that the proportion of apoptotic CCECs was markedly increased in both the HG group and the Aspartame group compared with the NC group (Figure 6E). In the NC group, the total apoptotic rate was low, whereas it increased substantially after high-glucose or aspartame treatment. Specifically, the total apoptotic rate, calculated as the sum of early apoptotic and late apoptotic cells, was approximately 1.62% in the NC group, 8.18% in the HG group, and 7.57% in the Aspartame group. After saxagliptin intervention, the apoptotic rate in the Aspartame + Saxagliptin group decreased to approximately 3.49%.

Consistent with the apoptosis assay results, flow cytometric detection of caspase-3 showed that the proportion of caspase-3-positive cells was markedly elevated in both the HG group and the Aspartame group compared with the NC group (Figure 6F). The percentage of caspase-3-positive cells was 2.05% in the NC group, 11.2% in the HG group, and 10.7% in the Aspartame group, whereas it decreased to 4.83% after saxagliptin treatment.

## Discussion

Aspartame is one of the most widely used non-nutritive sweeteners and is commonly found in sugar-free beverages and other low-calorie foods (1). Although current regulatory assessments generally regard aspartame as safe within approved usage ranges, accumulating evidence suggests that, under certain exposure conditions, aspartame may be associated with biological alterations such as oxidative stress, inflammatory responses, and vascular dysfunction (5, 29). Therefore, beyond its role as a sugar substitute, the potential health effects of aspartame warrant further investigation.

It should be noted that aspartame is rapidly hydrolyzed *in vivo* into phenylalanine, aspartic acid, and methanol (3). Therefore, the biological effects observed after aspartame exposure may not be solely attributable to the intact parent compound, but may also involve its major metabolites and downstream metabolic responses. These metabolism-related effects may contribute to oxidative stress, inflammatory activation, endothelial injury, and apoptosis (5, 7). Accordingly, the docking and molecular dynamics results should be interpreted as theoretical evidence suggesting a possible interaction between the parent structure of aspartame and DPP4, rather than definitive evidence that intact aspartame directly binds to DPP4 *in vivo*.

DPP4 is a widely expressed serine exopeptidase that exists both on the cell membrane and in a soluble circulating form (30). Traditionally, DPP4 has been mainly recognized as a glucose metabolism related enzyme because it cleaves substrates such as GLP-1, GIP, and SDF-1α (31). However, increasing evidence has shown that DPP4 is also deeply involved in pathological processes related to endothelial homeostasis imbalance, oxidative stress, and inflammation-associated vascular injury (32). DPP4 inhibition can increase NO bioavailability, improve endothelium-dependent vasodilation, and exert vasoprotective effects through the Akt/eNOS related signaling pathway (33). Several experimental studies have further demonstrated that different DPP4 inhibitors can promote eNOS activation and NO release, thereby improving endothelial cell function and post-ischemic revascularization (34, 35). In contrast, soluble DPP4 has been shown to directly induce microvascular endothelial dysfunction and impair acetylcholine-mediated endothelium-dependent relaxation, suggesting that excessive DPP4 activation may promote endothelial injury (36). In the present study, the wound healing assay showed that, compared with the normal control group, the repair capacity of penile corpus cavernosum endothelial cells was markedly reduced at 48 h after aspartame treatment, and the overall trend was similar to that observed in the high-glucose group. Western blot analysis further showed increased DPP4 expression and decreased p-eNOS levels in the Aspartame group, both of which were partially reversed after saxagliptin treatment.

In addition to its effect on eNOS/NO signaling, DPP4 is also closely related to apoptosis (34). Previous studies have shown that, under metabolic stress conditions such as hyperglycemia and high-fat exposure, abnormal activation of DPP4 is closely associated with endothelial injury and increased apoptosis (37, 38). DPP4 inhibition can attenuate high glucose-induced ROS production, mitochondrial membrane potential loss, and apoptosis, and its protective effect may be related to activation of AMPK signaling (39, 40). In high-fat diet-fed rats and palmitate-induced endothelial injury models, DPP4 inhibition has also been shown to suppress activation of the ROS–ER stress–CHOP axis, downregulate Bax, and upregulate Bcl-2, thereby inhibiting apoptosis (37). In addition, in diabetic ED models, saxagliptin has been reported to reduce DPP4 expression, enhance SDF-1/PI3K/AKT signaling, and improve cavernosal endothelial dysfunction, oxidative stress, and apoptosis, suggesting that DPP4 may participate in the pathogenesis of ED by regulating the balance between pro-survival and pro-apoptotic signaling pathways (28). Consistent with these previous findings, the present study found that aspartame treatment markedly increased the expression of the pro-apoptotic protein Bax while decreasing that of the anti-apoptotic protein Bcl-2, and the overall trend was similar to that observed in the high-glucose group. After saxagliptin treatment, Bax levels decreased and Bcl-2 expression was partially restored.

From a nutritional perspective, aspartame should be distinguished from other non-nutritive sweeteners, such as steviol glycosides, sucralose, and saccharin, because these compounds differ in their sources, metabolic profiles, ADI values, and potential biological effects (41). For example, steviol glycosides are generally derived from *Stevia rebaudiana*, and their ADI is usually expressed as steviol equivalents at 4 mg/kg body weight/day (42). Previous safety evaluations have not identified clear evidence of reproductive or developmental toxicity for steviol glycosides complying with JECFA specifications. By contrast, aspartame is rapidly hydrolyzed *in vivo* into phenylalanine, aspartic acid, and methanol, and its potential biological effects may be more closely related to oxidative stress, inflammatory responses, mitochondrial dysfunction, and apoptosis (5). Therefore, the findings of the present study should be regarded as aspartame-specific. In the absence of direct comparative evidence, these results should not be directly generalized to steviol glycosides or other sweeteners.

This study has several limitations. First, part of the evidence was derived from network toxicology, molecular docking, and molecular dynamics simulations. These approaches are computational and hypothesis generating in nature, and therefore cannot directly establish causal relationships or confirm direct interactions *in vivo*. Second, the transcriptomic analysis was based on the GSE2457 dataset, which has a relatively small sample size and may introduce potential bias or overfitting risk. Third, although preliminary concentration-gradient experiments were performed at the mRNA level, the subsequent functional assays and protein-level validation were mainly conducted using a single concentration and short-term *in vitro* exposure. Therefore, these experiments cannot fully recapitulate the complex regulatory environment *in vivo* or the long-term and repeated dietary exposure patterns encountered in real life. The dose–response relationship and overall impact of aspartame on ED still require further validation using long-term exposure models and animal experiments. Fourth, although DPP4, p-eNOS/eNOS, and Bax/Bcl-2 were examined, DPP4 enzymatic activity, NO/cGMP signaling, and DPP4-specific genetic intervention were not assessed, which limits the mechanistic interpretation of the present findings. Future studies should include reactive oxygen species ROS detection, inflammatory cytokine measurement, NO/cGMP level assessment, and further validation of the related signaling pathways. Fifth, although rat genes were converted to their human homologs, potential bias caused by interspecies differences cannot be completely avoided. Finally, aspartame is rapidly hydrolyzed *in vivo* into phenylalanine, aspartic acid, and methanol. The present study did not separately evaluate the effects of these metabolites on DPP4/eNOS signaling or endothelial dysfunction. Further studies are therefore needed to distinguish the relative contributions of the parent compound and its metabolites.

## Conclusion

The present study suggests that aspartame may participate in the development and progression of ED through a DPP4 related mechanism. Integrating network toxicology, molecular docking, molecular dynamics simulation, and *in vitro* experiments, we found that aspartame upregulated DPP4 expression, inhibited the migration of corpus cavernosum endothelial cells, decreased p-eNOS/eNOS and Bcl-2 expression, and increased Bax expression. Notably, the DPP4 inhibitor saxagliptin partially reversed these alterations, suggesting that aspartame may aggravate ED by promoting endothelial dysfunction and apoptosis. DPP4 may therefore represent a potential key target in the mechanism underlying aspartame related ED.

## Data Availability

The datasets presented in this study can be found in online repositories. The names of the repository/repositories and accession number(s) can be found in the article/supplementary material.
